# Hemodynamic changes after spinal anesthesia in preeclamptic patients undergoing cesarean section at a tertiary referral center in Ethiopia: a prospective cohort study

**DOI:** 10.1186/s13037-020-00234-w

**Published:** 2020-03-31

**Authors:** Tikuneh Yetneberk Alemayehu, Yophetah Woldegerima Berhe, Habtamu Getnet, Mamaru Molallign

**Affiliations:** 1 Department of anesthesia, Debre Tabor University, Debre Tabor, Ethiopia; 2 Department of anesthesia, Univesity of Gondar, Gondar, Ethiopia

**Keywords:** Preeclampsia, Spinal anesthesia, Cesarean section, Hypotension

## Abstract

**Background:**

Spinal anesthesia-induced maternal hypotension is the most frequent complication associated with maternal morbidity and mortality during Cesarean section. The aim of this study was to compare the incidence and magnitude of hemodynamic changes in preeclamptic and non-preeclamptic parturients undergone Cesarean section under spinal anesthesia.

**Method:**

A prospective cohort study was conducted from 01 February to 28 May 2019 in preeclamptic and non-preeclamptic parturients. We hypothesized preeclamptic parturients are at high risk of spinal anesthesia induced hypotension than non preeclamptics. A total of 122 ASA II and ASA III parturients were recruited consecutively and assigned to two groups (81non-preeclamptics, and 41 preeclamptics). Parturients with cardiac disease, twin pregnancy, chronic hypertension, gestational hypertension, superimposed hypertension, renal disease, diabetes mellitus, coagulopathy (platelet count < 80 × 10^9^/L), active labor, eclampsia, abruptio placentae, placenta praevia, any adjuvant added with local anesthetics were excluded. The data analysis was done using SPSS version 22 statistical software. Student t test, MannWhitney U test and Fisher exact test were used to compare the data. All *P* values < 0.05 were considered statistically significant.

**Result:**

The incidence of spinal anesthesia-induced hypotension was higher in non-preeclamptic parturients than preeclamptic parturients (55.6% vs. 34.1%, respectively) and the degree of blood pressure drop was significantly greater in the non-preeclamptic parturients compared to those with preeclampsia; As well intraoperative fluid consumption was significantly greater in the non-preeclamptics parturients compared to those with preeclamptics.

**Conclusion:**

The incidence and magnitude of spinal anesthesia-induced hypotension in parturients undergone Cesarean section were less in preeclamptic parturients than in non-preeclamptic parturients.. Based on the data from this study we recommended spinal anesthesia for preeclamptic patients, unless there is a contra indication based on preeclampsia.

## Background

Worldwide preeclampsia/eclampsia is the third leading cause of maternal morbidity and mortality [1]. This is especially the commonest cause of fetomaternal complications in developing countries; around 40 to 60% of maternal deaths in these countries are caused by preeclampsia alone; in Ethiopia,19% of maternal mortality is caused by hypertensive disorder of pregnancy [2]. The definitive management of preeclampsia is delivery where Cesarean section is commonly promoted [3]. A study done in Ethiopia showed that 6.1% of indications for CS were preeclampsia [4]. There is a paradigm shift in the practice of obstetrics anesthesia from general to spinal anesthesia for Cesarean section, but the rate of preventable spinal anesthesia-related deaths is still high [5–7]. Hypotension after spinal anesthesia was the most frequent complication associated with maternal morbidity and mortality during Cesarean section [8]. Preeclamptic patients have been considered to be at higher risk of profound hypotension when they undergo Cesarean section under spinal anesthesia [9].

A study had shown that fluid loading and vasopressor prophylaxis were effective in reducing the incidence of spinal anesthesia-induced hypotension in healthy parturients [10]. But these preemptive measures could put the preeclamptic patients at increased risk of hypertension and pulmonary edema [9]. Due to inconsistent definition, the reported incidence of spinal anesthesia-induced maternal hypotension varies between 7 and 89.2% [11, 12]. Therefore, anesthetic management for preeclamptic parturients who undergo Cesarean section is challenging for anesthetists [13]. To alleviate maternal deaths arise from risks attributable to pregnancy and childbirth, the provision of safe anesthesia is necessitating [3, 14, 15].

During obstetric anesthesia, preservation of hemodynamic stability is a big concern for anesthetists [13], especially for preeclamptic parturients who planned to undergo Cesarean section [12, 13]. However, spinal anesthesia-induced maternal hypotension is still the most frequent complication [16, 17]. Anesthetists denied spinal anesthesia for preeclamptic parturients, due to the fear of profound hypotension and its management crisis (exaggerated response to vasopressor treatment and pulmonary edema following fluid challenges) [9, 13, 18–20]. Furthermore, the incidence of spinal anesthesia-induced maternal hypotension showed inconsistency across different studies, which makes it almost difficult to set standard targets and develop a local management protocol [16, 17, 21].

## Methods

A prospective observational cohort study was conducted to determine the incidence of hypotension and the magnitude of hemodynamics change following spinal anesthesia in preeclamptic and non-preeclamptic parturients undergone Cesarean section in University of Gondar comprehensive specialized hospital; Northwest Ethiopia from 01 February to 28 May 2019. We hypothesized preeclamptic parturients are at high risk of spinal anesthesia induced hypotension than non preeclamptics. ASA II and ASA III parturients were involved in the study. Parturients with cardiac disease, twin pregnancy, total spinal, chronic hypertension, gestational hypertension, superimposed hypertension, renal disease, diabetes mellitus, coagulopathy (platelet count < 80 × 10^9^/L), active labor, eclampsia, abruptio placentae, placenta praevia, any adjuvant added with local anesthetics were excluded. Variables like age, height, BMI, ASA status, gestational age, and amount of fluid preloaded, amount of fluid consumed intraoperatively, the weight of the neonate, upper sensory level of the spinal block at the time of skin incision, position during and after the spinal procedure were studied.

### Intraoperative hypotension

Defined as more than 20% decrease in the mean arterial blood pressure following spinal anesthesia compared to the baseline in both groups [16, 17, 21, 22]. Preeclampsia: A pregnancy-induced increase in blood pressure ≥ 140/90 mmHg after 20 weeks of gestation and proteinuria ≥300 mg/24 h [12]. Preeclampsia with severity feature/severe preeclampsia: Defined as a systolic arterial blood pressure of 160 mmHg or higher, or a diastolic blood pressure of 110 mmHg or higher, associated with proteinuria > 5 g in 24 h [12, 23]. Change in heart rate: When there was a 20% decrease or increase in heart rate from baseline [6, 12, 22, 24, 25]. The magnitude of hemodynamics change/severity of hypotension: The percentage falls of blood pressure (SBP, DBP, and MAP) between two measurements and it was calculated as [12, 22, 26]:

$$ \mathrm{Percentage}\kern0.34em \mathrm{fall}=\frac{\mathrm{baseline}\kern0.34em \mathrm{measurement}\kern0.34em \mathrm{value}-\mathrm{current}\kern0.34em \mathrm{measurement}\kern0.34em \mathrm{value}}{\mathrm{baseline}\kern0.34em \mathrm{measurement}\kern0.34em \mathrm{value}}\times 100 $$

All consecutive parturients who fulfilled the inclusion criteria and gave birth by Cesarean section under spinal anesthesia were included in the study until the intended sample size has been achieved. The sample size was determined based on the latest study done in Macedonia the incidence of hypotension in preeclamptic and non-preeclamptic parturients was 25 and 53% respectively [22] and calculated by using the Fleiss correction factor method with a power of 80% at a 5% significance level and the sample size was 122. Hereafter, 41 participants were enrolled in the preeclamptic group and 81 participants were enrolled in non-preeclamptic groups with a proportion of 1:2 ratios respectively. All parturients who satisfy the inclusion criteria were included in the study. There was homogeneity of variance, as assessed by Levene’s test for equality of variances.

In the operation theater, baseline hemodynamic variables (SBP, DBP, MAP, and HR) were recorded. Baseline BP was taken as the mean of the two readings measured 1 min apart and 5 min after the parturient arrived in the operation theatre and before doing any invasive procedures. After spinal anesthesia SBP, DBP, MAP, and HR were recorded every 2 min for 30 min and every 5 min thereafter until the end of surgery. Patients were monitored with non-invasive automated blood pressure cuffs, ECG, and pulse oximetry. The data collectors have assessed the upper level of sensory block bilaterally by pinprick at the time of skin incision and it was documented. The total intraoperative fluid consumption, total estimated blood loss, the weight of the newborn were documented as well. The data collection technique was a combination of chart review, observation, and interview using a pre-tested questionner that was developed in English language.

After completion of the data collection, the data was entered into Epidata version 4.2. Software and exported to SPSS version 22 statistical software for further analysis. The data were tested for normality with Shapiro Wilk U-test and normally distributed data were compared by using the independent student’s t-test and expressed as mean ± SD. Whereas non-normally distributed data were compared using the Mann-Whitney U- test and expressed as medians (IQR). Fisher’s exact test was used for intergroup comparisons of proportion. All *P* values < 0.05 were considered statistically significant. The research was taken ethical clearance from University of Gondar College of medicine and health science ethical review board and written informed consent was taken from each study participants.

## Results

A total of 122 parturients were enrolled (81 non-preeclamptic and 41 preeclamptic parturients) in this study. There were no statistically significant differences in socio-demographic and anesthetic characteristics of parturients such as; age, weight, height, the volume of 0.5% plain bupivacaine, and speed of spinal administration between groups (Tables 1 and 2). The majority of preeclamptic parturients were ASA II and the remains were ASA III, while, All parturients in the non-preeclamptic group were ASA II, and this difference was statistically significant between groups; *p* <  0.001(Table 1). The mean gestational age at the time of Cesarean section was significantly lower in the preeclamptic group: 38.56 ± 1.63 weeks in non-preeclamptic versus 37.44 ± 1.25 weeks in preeclamptics; *p* = 0.001(Table 1). However, there was no statistically significant difference in the mean weight of the newborn between groups; *p* = 0.37 (Table 1).

**Table 1:** Maternal and neonatal characteristics, University of Gondar Northwest Ethiopia, May 2019(*n* = 122)

Variable	non-preeclampsia(*n* = 81)	Preeclampsia(*n* = 41)	*p*-value
Age (year)^a^	27.93 ± 3.60	27.95 ± 3.99	0.972
Weight (kg)^a^	64.72 ± 7.82	65.95 ± 7.65	0.408
Height (cm)^a^	162.47 ± 6.29	164.10 ± 5.83	0.169
BMI (kg/m^2^)^a^	24.63 ± 3.57	24.57 ± 3.18	0.927
ASA status n (%)			< 0.001
ASA II	81(100)	28(68.3)	
ASA III		13(31.7)	
Nulliparous n (%)	33(40.7)	21(51.2)	0.335
Gestational age (week)^a^	38.56 ± 1.63	37.44 ± 1.25	0.001
weight of the new born (kg)^a^	3.03 ± 0.42	2.96 ± 0.40	0.374
Previous cesearn section n (%)			0.293
Yes	26(32.1)	9(22)	
No	55(67.9)	32(78)	

*n* number, *Kg* kilogram per meter square, *cm* centimeter, *ASA* American society of anesthesiologists, *BMI* body mass index

^a^Independent student t-test

The median upper sensory level at the time of skin incision was higher in the preeclamptic parturients compared to those with non-preeclamptics and this difference was statistically significant (T5 vs. T6; *p* = 0.032) (Table 2). The baseline SBP, DBP, MAP, and heart rate were higher in parturients with preeclampsia than the corresponding values among the non-preeclamptic parturients (Table 3). Non-preeclamptic parturients have been taken a higher volume of preload fluid compared with preeclamptics (611.67 ml ± 289.65 VS 565.44 ml ± 318.45; *p* = 0.004) (Table 4) and there was a statistically significant difference in intraoperative intravenous fluid consumption between groups, which was higher in non-preeclamptics compared to preeclamptic parturients (1723.46 ml ± 352.41vs1463.41 ± 417.59; *p* = 0.001) Table 4). The mean duration of surgery was comparable between the two groups (Table 4).

**Table 2:** Anesthetic characteristics and procedural position of parturients; University of Gondar Northwest Ethiopia, May 2019 (*n* = 122)

Variable	Non-preeclamptic (*n* = 81)	Preeclamptic(*n* = 41)	*p*-value
Volume of injected bupivacaine (ml)^a^	2.30 ± 0.25	2.27 ± 0.25	0.558
Dose of 0.5% plain bupivacaine (mg)^a^	11.48 ± 1.24	11.34 ± 1.26	0.558
Speed of spinal administration (ml/sec)^a^	0.18 ± 0.12	0.21 ± 0.08	0.323
Upper sensory level^b^	T6(T4-T6)	T5(T4-T6)	0.032
Position during spinal procedure n (%)			0.223
Sitting	80(98.8)	39(95.1)	
Lateral	1(1.2)	2(4.9)	
Position after spinal procedure n (%)			0.479
Supine	80(98.8)	41(100)	
Left Lateral tilt	1(1.2)		
Parturients treated with adrenaline intraoperatively n (%)			0.550
Yes	2(2.5)	41(100)	
No	79(97.5)		

*n* Number, *SA* Spinal anesthesia, *SBP* Systolic blood pressure, *DBP* Diastolic blood pressure, *MAP* Mean arterial pressure, *mg* Milligram, *ml* Milliliter, *ml/sec* Milliliter per second, *IV* Intravenous

^a^Independent student t-test. ^b^Mann-Whitney U-test

**Table 3:** Baseline hemodynamic characteristic of the parturients, University of Gondar Northwest Ethiopia, May 2019 (*n* = 122)

Variable	non-preeclampsia(*n* = 81)	Preeclampsia(*n* = 41)	*p*-value
Baseline SBP (mmHg)	118.83 ± 9.22	134.95 ± 11.71	0.001
Baseline DBP (mmHg)	75.52 ± 8.64	85.90 ± 10.40	0.001
Baseline MAP (mmHg)	83.20 ± 8.45	85.32 ± 10.24	0.218
Baseline heart rate (beats/minute)	95.95 ± 15.79	99.38 ± 20.21	0.321

Independent student t-test

*SBP* Systolic blood pressure, *DBP* Diastolic blood pressure, *MAP* Mean arterial pressure, *mmHg* Millimeter mercury

**Table 4:** Fluid consumption, estimated blood loss and surgical conditions, University of Gondar Northwest Ethiopia, May 2019 (*n* = 122)

Variable	non-preeclampsia(*n* = 81)	Preeclampsia(*n* = 41)	*p*-value
crystalloid preload (ml)	611.67 ± 289.65	565.44 ± 318.45	0.004
Intraoperative IV fluid (ml)	1723.46 ± 352.41	1463.41 ± 417.59	0.001
Estimated blood loss (ml)	382.96 ± 134.12	379.02 ± 132.74	0.878
Duration of surgery (minute)	43.89 ± 11.75	42.68 ± 9.16	0.567
Experience of obstetrician (year)	2.93 ± 0.67	3.00 ± 0.84	0.596
Experience of anesthetist (year)	3.42 ± 1.39	3.71 ± 1.27	0.268

Independent student t-test

*n* Number, *SA* Spinal anesthesia, *SBP* Systolic blood pressure, *DBP* Diastolic blood pressure, *MAP* Mean arterial pressure, *ml* Milliliter, *IV* Intravenous

**Fig. 1:**
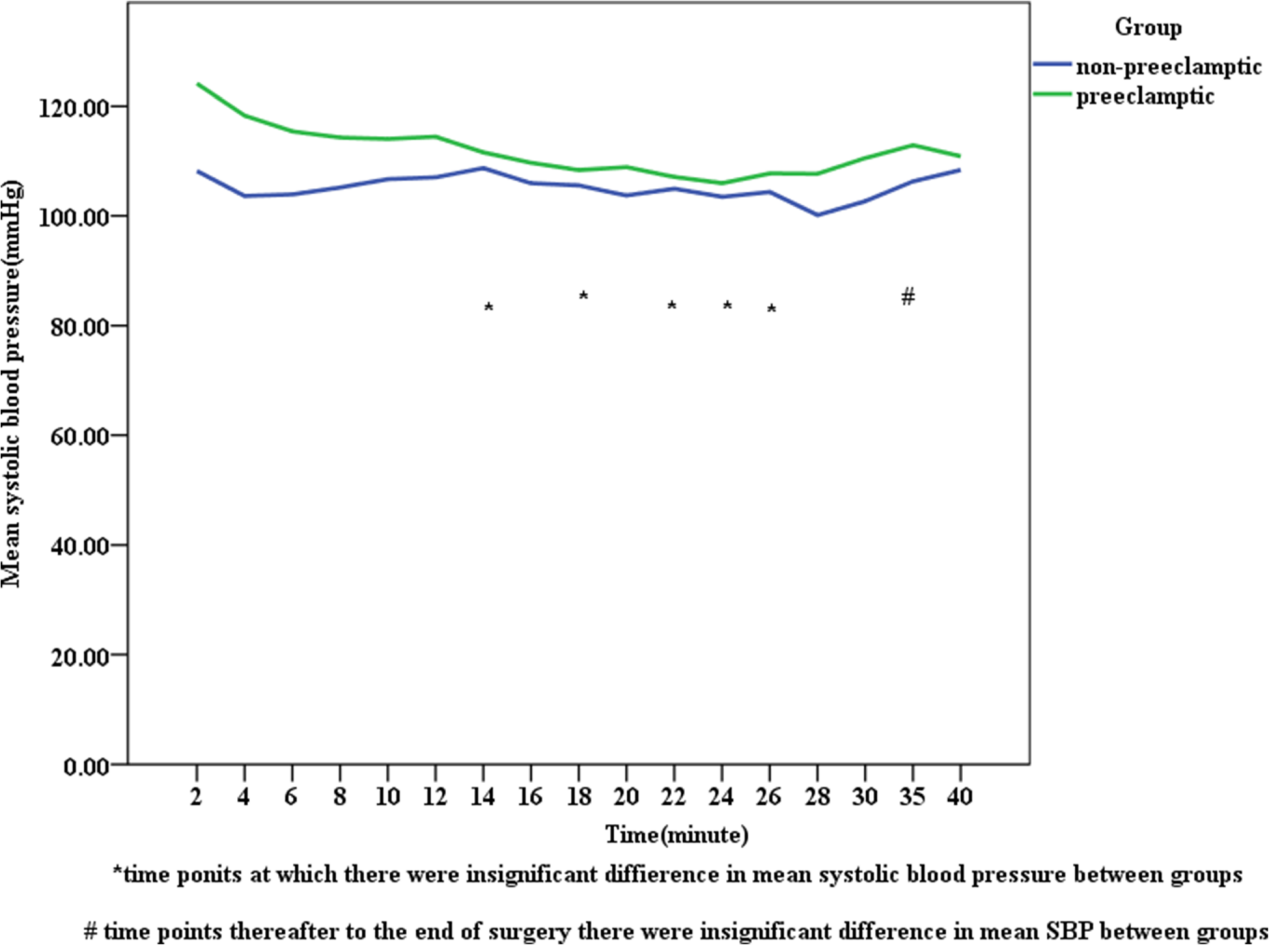
Trends of mean arterial blood pressure change following spinal anesthesia in non-preeclamptic and preeclamptic parturients, University of Gondar Northwest Ethiopia, May 2019 (*n* = 122)

**Fig. 2:**
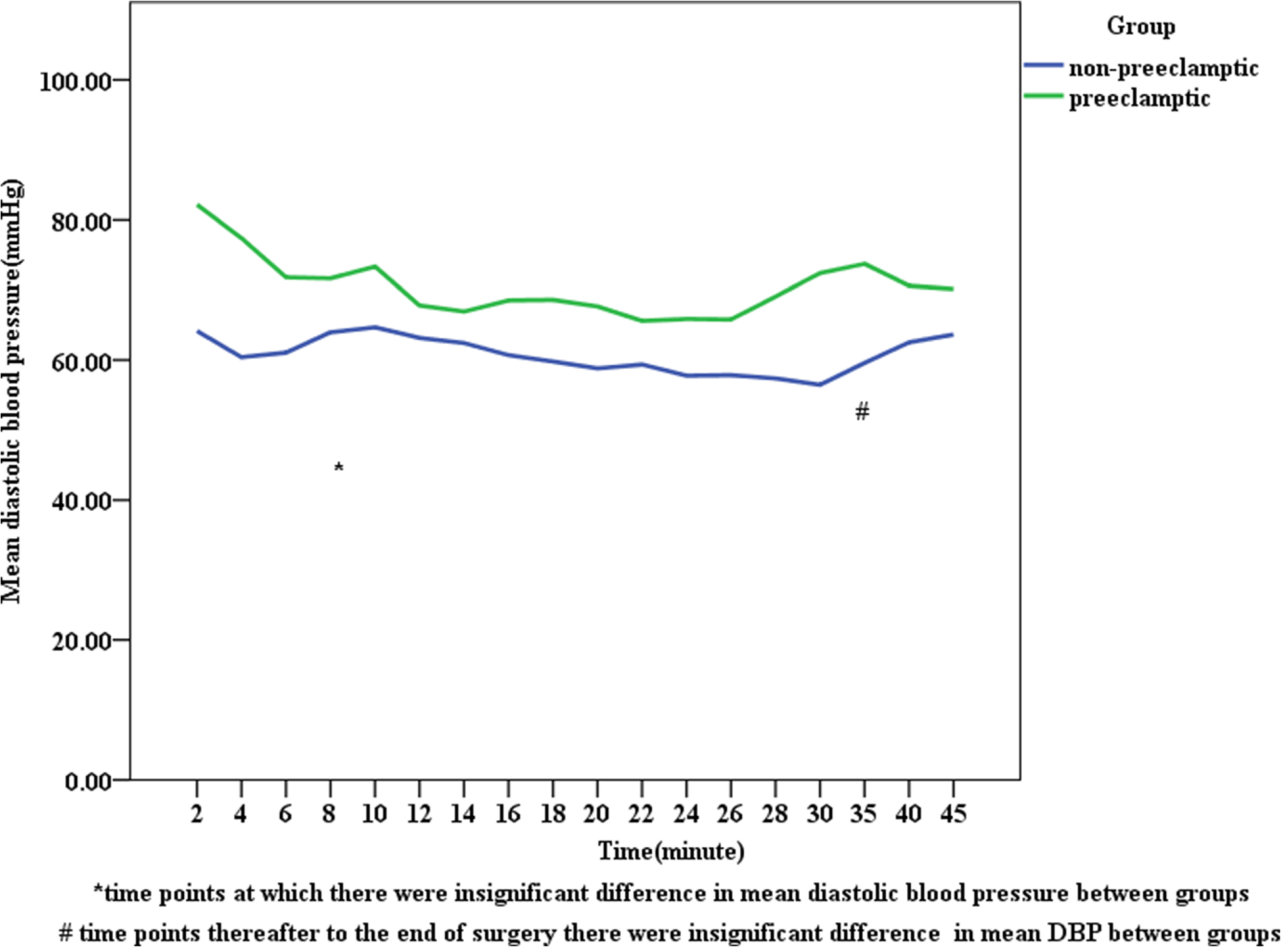
Trends of systolic blood pressure change following spinal anesthesia in non-preeclamptic and preeclamptic parturients, University of Gondar Northwest Ethiopia, May 2019 (*n* = 122)

**Fig. 3:**
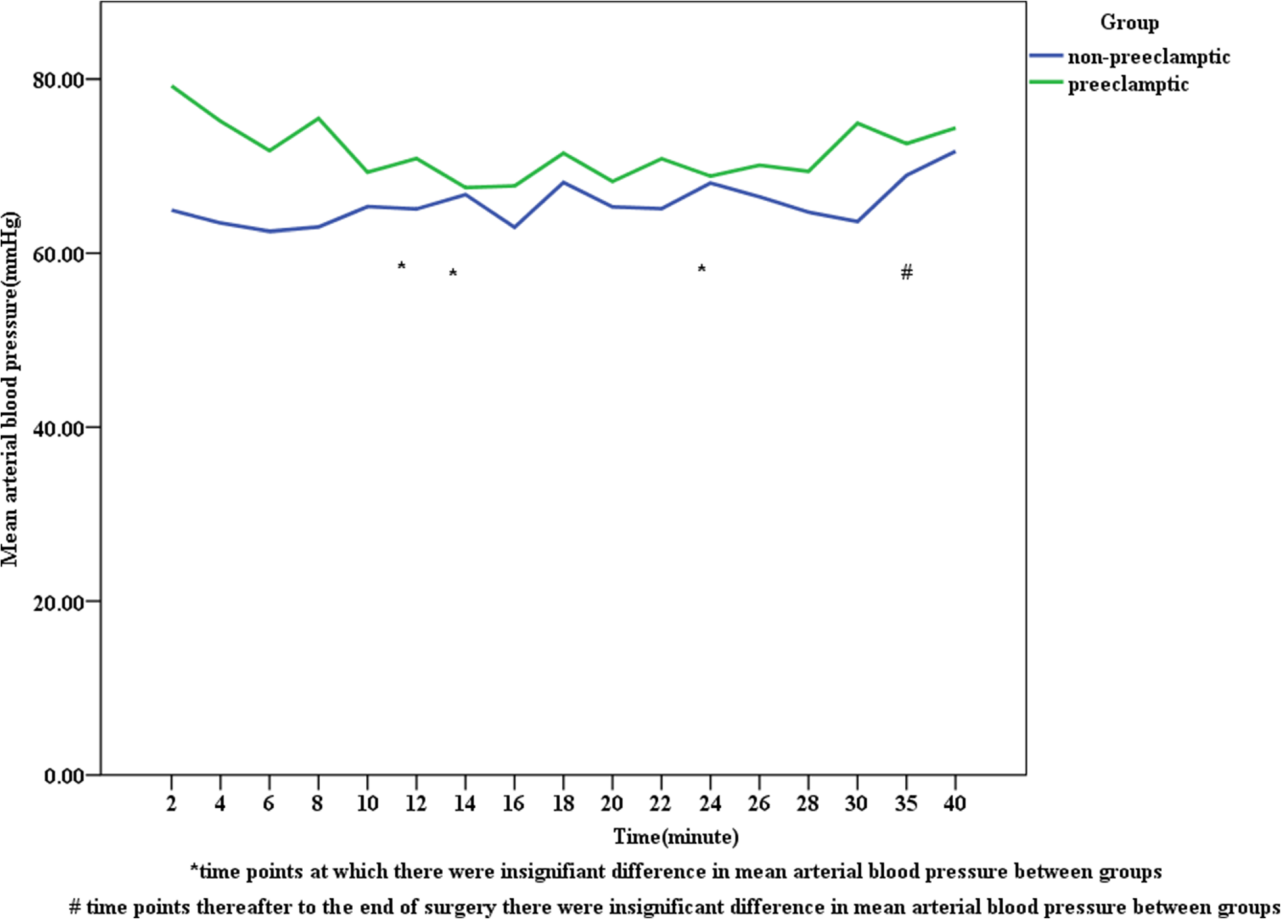
Trends of diastolic blood pressure change following spinal anesthesia in non-preeclamptic and preeclamptic parturients, University of Gondar Northwest Ethiopia, May 2019 (*n* = 122)

In the preeclamptic parturients, mean SBP and DBP were higher than the corresponding values among non-preeclamptic parturients following spinal anesthesia at each point of time (Figs. 1 and 2) and the same fashion was happening to MAP, which was at a higher level in preeclamptic parturients than non-preeclamptic parturients (Fig. 3). The incidence of hypotension in non-preeclamptic parturients (55.6%) was higher than that of preeclamptic parturients (34.1%) (Table 5), despite the former receiving much volume of intravenous fluid (1723.46 ml ±352.41 versus 1463.41 ml ±417.59; *p* = 0.001) (Table 4). There was also a decrease in blood pressure after spinal anesthesia in both groups, but the magnitude of blood pressure falls were significantly greater in the non-preeclamptic parturients compared to those with preeclampsia: 27.78% ± 5.44 vs. 21.05% ± 3.06 for SBP, 26.18% ± 4.07 vs. 23.93% ± 4.79 for DBP, and 25.65% ± 2.22 vs. 21.27% ± 15.15 for MAP (*p* < 0.001) (Table 5).

**Table 5:** Incidence and magnitude of hemodynamic changes following spinal anesthesia, University of Gondar Northwest Ethiopia, May 2019 (*n* = 122)

Variable	Non-preeclampsia(*n* = 81)	Preeclampsia(*n* = 41)	*p*-value
Incidence of hypotension n (%) ^b^	45(55.6)	14(34.1)	0.035
Lowest SBP after SA (mmHg)	85.5 ± 2.12	106.80 ± 12.17	
Decrease from baseline %^a^	27.78 ± 5.44	21.05 ± 3.06	< 0.001
lowest DBP after SA (mmHg)	56 ± 8.55	65 ± 5	
Decrease from baseline %^a^	26.18 ± 4.07	23.93 ± 4.79	< 0.001
lowest MAP after SA (mmHg)	61.99 ± 7.59	65 ± 0.00	
A decrease from baseline %^a^	25.65 ± 2.22	21.27 ± 15.15	< 0.001
Mean HR after SA (beats/minute)^a^	90.40 ± 9.96	89.21 ± 12.33	0.567
20% decrease in HR n (%)	72(88.9)	31(75.6)	0.068
20% increase in HR n (%)	2(2.5)		0.550

*n* Number, *SA* Spinal anesthesia, *SBP* Systolic blood pressure, *DBP* Diastolic blood pressure, *MAP* Mean arterial pressure, *HR* Heart rate, *mmHg* Millimeter mercury

^a^Independent student t-test. ^b^ Fisher’s exact test

## Discussion

During Cesarean section, hypotension following spinal anesthesia was the commonest complication related to maternal morbidity and mortality [6, 8, 22]. Because of inconsistent definitions, the reported incidence of hypotension after spinal anesthesia in Cesarean section varies between 7 and 89.2% [6, 8, 10–12]. There was a widespread belief that preeclamptic parturients were considered at higher risk of profound hypotension following spinal anesthesia [12, 13, 22, 27]. This concern may often frighten anesthetists from choosing spinal anesthesia for Cesarean section in preeclamptic parturients [12, 13, 22].

Nikooseresht M et al. found that SBP, DBP, and MAP measured at the baseline were higher for the patients with preeclampsia, and the lowest mean SBP, DBP, and MAP measured among the preeclamptic patients were higher than the corresponding values among the healthy parturients [12]. This finding was in line with our study result. In this study, the incidence of hypotension after spinal anesthesia in preeclamptic parturients (34.1%) was less than that of non-preeclamptic parturients (55.6%) (*p* = 0.035). The discrepancy in the incidence of hypotension related to preeclampsia related factors. Despite the sympathetic block due to spinal anesthesia, because of exaggerated vasoconstriction, preeclamptic parturients can still maintain their vascular tone that caused only a limited decrease in blood pressure.

Following spinal anesthesia, the mean SBP, DBP, and MAP measured at different time points were higher in preeclamptic parturients than the corresponding values among non-preeclamptic parturients. But this difference was insignificant between groups at 14, 18, 22, 24, 26,35 min in SBP, at 8 and 40 min in DBP, at 10, 14, 24, 35 min in MAP, and thereafter to the end of surgery. Whereas, the mean pulse rate was comparable between groups at different time points after SA. Mitra M et al. found significant differences in SBP, DBP, and MAP at each point of time in both groups [26]. The possible explanation for this discrepancy might be the employment of invasive blood pressure monitoring in their study, in contrast to our study.

Similar to our study Aya AG et al. found that severely preeclamptic patients had a less frequent incidence of clinically significant hypotension compared to healthy parturients (16.6% versus 53.3%;*P* = 0.006 [28]. The incidence of hypotension among preeclamptic parturients in our study was higher than Aya AG et al. result. The likely reason may be the use of different criteria for defining hypotension (20% versus 30% decline to baseline MAP) and the use of the small volume of preload in our study participants compared to Aya AG et al. (565.38 ml ± 318.4 vs1653 ml ± 331).

In contradict to our result, Mendes et al. found that there was no statistically significant difference regarding the occurrence of hypotension after spinal anesthesia between severely preeclamptic and healthy parturients. But the incidence rate of hypotension was high in both groups (84and70%,*p* = 0.45) [25]. This difference may be due to the intraoperative administration of intravenous hydralazine in preeclamptic parturients in their study.

Sivevski A et al. found that the percentage of fall of BP from baseline were significantly greater in the healthy parturients compared to those with preeclampsia (25.8% ± 10.1 vs. 18.8% ± 17.0 for SBP, 28.5% ± 8.8 vs. 22.5% ± 10.4 for DBP, and 31.2% ± 14.2 vs. 18.2% ± 12.6% for MAP, *p* < 0.05 [22]. Likewise, another study conducted by Saha D et al. found that the percentage of fall of DBP and MAP calculated from the baseline was also less in the preeclamptic group (34.5 and 33% in normotensive as opposed to 30.3 and 32.3% in preeclamptics, respectively) [1]. The result of our study was in accordance with the above findings.

Unlike our study, Mendes et al. found that there was no significant difference in the lowest mean drop of SBP and DBP after spinal anesthesia between preeclamptic and healthy parturients [25]. This difference may be due to standardized fluid management and administration of potent direct vasodilator during surgery (intravenous hydralazine) in preeclamptic parturients in their study. In this study, a decreasing dose of 0.5% bupivacaine was practiced for the Cesarean section. However, the incidence of hemodynamic change had not a significant difference (10 mg versus 12.5 mg). This finding was corresponding with a study done by Moshiri E et al. [29]. The result of our study showed that the mean gestational age in parturients with preeclampsia was considerably different compared with those of the non-preeclamptic parturients. This finding was in line with a study done by Sivevski A et al. [22].

Comparable to Sivevski A et al. finding [22], the result of our study showed that there was a statistically significant difference regarding the volume of preload taken between groups, which was higher in non-preeclamptic parturients compared to preeclamptic parturients (611.67 ml ± 289.65 VS 565.44 ml ± 318.45; *p* = 0.004). In our study, intraoperative fluid consumption was lower in preeclamptic parturients compared with non-preeclamptic parturients (1463.41 ± 417.59 VS 1723.46 ml ± 352.41; *p* = 0.001). This result was in line with Nikooseresht M et al. [12]. Similar to a study done by Lavie A et al. [24], in our study, the total estimated blood loss was comparable between groups, and no blood products were required throughout the procedure. Nikooseresht M et al. also found that the surgical durations were comparable between two groups [12]. This finding was in line with our study result.

In our study measurement of vasopressor consumption was difficult, due to the absence of standardized vasopressor usage in the hospital. Anesthetists were trying to manage hypotension with fluids and adrenaline accordingly. In this study, two parturients in the non-preeclamptic group were treated with adrenaline but there were no parturients treated with adrenaline in the preeclamptic groups. However, this difference was not statistically significant (*p* = 0.550). Even though our study does not quantify it, studies found that hypotension requiring vasopressor medication (ephedrine and phenylephrine) following spinal anesthesia was less common in parturients with preeclampsia than in non-preeclamptic parturients [13, 22, 26, 27, 30–32]. The limitation of this study was the small sample size, observational study design which was difficult to control all possible co-founders (like oxytocin), and inability to quantify vasopressor consumption; due to lack of standardized vasopressor (ephedrine and phenylephrine) usage in the practice, which could affect the trends of hemodynamic change over time. As well the use of non-invasive blood pressure measurement in this study might miss some data which can be noticed in invasive blood pressure measurement.

## Conclusion

This study showed that the incidence and magnitude of spinal anesthesia-induced hypotension in parturients undergone Cesarean section were less in preeclamptics than in non-preeclamptic parturients. In the preeclamptics group, they also experienced spinal anesthesia-induced hypotension, but the incidence and degree of hypotension were significantly lower than non-preeclamptic parturients.

## Data Availability

All data generated or analysed during this study are included in this published article.

## Abbreviations

ASA: American Society of Anesthesiologists
BMI: Body Mass Index
BP: Blood Pressure
CS: Cesarean Section
DBP: Diastolic Blood Pressure
GA: General Anesthesia
MAP: Mean Arterial Pressure
SA: Spinal Anesthesia
SBP: Systolic Blood Pressure
SD: Standard Deviation
